# Genetic Structure and TALome Analysis Highlight a High Level of Diversity in Burkinabe *Xanthomonas Oryzae* pv. *oryzae* Populations

**DOI:** 10.1186/s12284-023-00648-x

**Published:** 2023-07-31

**Authors:** A. Diallo, I. Wonni, A. Sicard, L. Blondin, L. Gagnevin, C. Vernière, B. Szurek, M. Hutin

**Affiliations:** 1 INERA, Institut de l’Environnement et de Recherches Agricoles du Burkina Faso, Laboratoire de Phytopathologie, Bobo-Dioulasso, Burkina Faso; 2 PHIM Plant Health Institute, Université de Montpellier, IRD, CIRAD, INRAE, Institut Agro, Montpellier, France

**Keywords:** Rice, Bacterial leaf blight, *Xoo*, TALE, Genotyping, Microsatellites, Molecular epidemiology

## Abstract

Bacterial Leaf Blight of rice (BLB) caused by *Xanthomonas oryzae* pv. *oryzae* (*Xoo*) is a major threat for food security in many rice growing countries including Burkina Faso, where the disease was first reported in the 1980’s. In line with the intensification of rice cultivation in West-Africa, BLB incidence has been rising for the last 15 years. West-African strains of *Xoo* differ from their Asian counterparts as they (i) are genetically distant, (ii) belong to new races and, (iii) contain reduced repertoires of Transcription Activator Like (TAL) effector genes. In order to investigate the evolutionary dynamics of *Xoo* populations in Burkina Faso, 177 strains were collected from 2003 to 2018 in three regions where BLB is occurring. Multilocus VNTR Analysis (MLVA-14) targeting 10 polymorphic loci discriminated 24 haplotypes and showed that *Xoo* populations were structured according to their geographical localization and year of collection. Considering their major role in *Xoo* pathogenicity, we assessed the TAL effector repertoires of the 177 strains upon RFLP-based profiling. Surprisingly, an important diversity was revealed with up to eight different RFLP patterns. Finally, comparing neutral vs. *tal* effector gene diversity allowed to suggest scenarios underlying the evolutionary dynamics of *Xoo* populations in Burkina Faso, which is key to rationally guide the deployment of durably resistant rice varieties against BLB in the country.

**Supplementary Information:**

The online version contains supplementary material available at 10.1186/s12284-023-00648-x.

## Introduction

Rice is a staple food for more than half of the world population, most of it living in developing countries. In Africa, where global food security is still a challenge, improving rice production to reach self-sufficiency is of major concern. In Burkina Faso, rice production has increased more than three-fold over the last 10 years (FAO). Accompanying this agricultural intensification is the risk of pathogens emergence, which could be devastating if no locally adapted control solutions are deployed (Gregory et al. 2009).

Among rice pathogens, *Xanthomonas oryzae* pv. *oryzae* (*Xoo*) is the causal agent of bacterial leaf blight (BLB), which is the most damaging bacterial disease of rice with up to 50% of yield losses (Niño-Liu et al. 2006). This vascular pathogen enters through the leaf via hydathodes or wounds and colonize the xylem vessels, leading to chlorotic lesions that mirror the progression of the bacteria along the leaf blade. Wind, contaminated water and human activities may favor disease transmission (Niño-Liu et al. 2006), while the role of seeds remains to be demonstrated. First described in Japan in 1884, BLB is today present in major rice producing countries in Asia and Africa.

Varietal resistance appears as the most effective way to control the disease considering environmental impact and cost (Ji et al. 2018). Extensive genetic and genomic studies have resulted in the identification of more than 40 resistance (*R*) genes since the discovery of the first BLB *R* gene *Xa1* (Ji et al. 2018). To date 12 *R* genes have been cloned and 9 of them depends of the “Transcription Activator Like Effector” (TALE) family, reflecting their crucial role in pathogenicity and the importance of gaining knowledge about TALEs to manage resistance (Ji et al. 2020; Jiang et al. 2020). TALEs are translocated in the plant host cells where they act as eukaryotic transcription factors able to induce genes by targeting specific promoter sequences called effector binding elements (EBE). EBE recognition is determined by the TALE’s central region composed of a variable number of nearly identical 33–35 amino-acid repeats, where residues in position 12 and 13, also named Repeat-Variable-Diresidues (RVDs), are highly variable and define DNA recognition specificity according to the TALE code (Boch et al. 2009; Moscou and Bogdanove 2009). TALEs N- and C-terminal regions are highly conserved across *Xoo* and harbor domains responsible for translocation by the Type III Secretion System, nuclear localization and transcriptional activation of target host genes. Some of these targets are named Susceptibility (*S*) genes and are required for full disease development (Garcia-Ruiz et al. 2021). *S* genes known to date mostly encode for either transcription factors or transporters, including clade III members of the SWEET family. Among them, *SWEET14* is induced by four TALEs targeting distinct EBEs. TalC and TalF are only present in African strains of *Xoo*, while AvrXa7 and PthXo3 are only found in Asian ones (Streubel, 2013). No other *SWEET* gene is known to be induced by African strains of *Xoo* (Doucouré et al. 2018; Oliva et al. 2019). Changes in the EBE of a major *S* gene may lead to resistance by loss of susceptibility (Hutin et al. 2015a), as illustrated by the recessive resistance genes *xa13*, *xa25*, and *xa41* that contain polymorphisms in the EBE of *SWEET11*, *SWEET13* and *SWEET14*, respectively (Zhou et al. 2015; Hutin et al. 2015a, b; Chu et al. 2006). TALE-mediated resistance may also result in the induction of so-called Executor (*E*) genes, which also possess EBEs in their promoter regions (Zhang et al. 2015). *Xa7*, *Xa10*, *Xa23* and *Xa27* are *E* genes induced by AvrXa7, AvrXa10, AvrXa23 and AvrXa27, respectively (Chen et al. 2021; Zhang et al. 2015). No *E* genes induced by African TALEs have been identified to date.

Strains of *Xoo* contain from 9 to 19 TALEs but their function in pathogen virulence is only known for one or two of them (Boch and Bonas 2010). It thus remains to be investigated why *Xoo* has so many TALEs, and how they contribute to virulence. *Xo* TALE repertoires (TALome) are organized in gene clusters along the chromosome and it is suggested that *tal* genes evolve by rearrangement of their repeat sequences, by mutation, and/or deletion of individual repeats (Erkes et al. 2017). Accordingly, some repeat arrays were reported between unrelated TALEs of a same strain, suggesting that intergenic recombination may have occurred to create new variants (Booher et al. 2015; Tran et al. 2018a). Because to their role in pathogenicity and the deployment of matching *R* genes in rice varieties, evolution of TALEs must be under constant selective pressure and thus rapidly evolving (Schandry et al. 2018). Knowing the diversity of TALE repertoires (TALomes) and understanding their evolution is then crucial to deploy varieties with locally adapted resistance genes and to better anticipate the risk of emergence of new aggressive strains.

Deployments of resistance (*R*) gene shape pathogen population structure by selecting strains that remain virulent (McDonald and Linde 2002). In the Philippines, where 10 races of *Xoo* have been described based on their phenotype on near isogenic lines containing a panel of *R* genes, the evolutionary history and changes in the prevalence of these races within populations were investigated. Using the genomic sequences of 10 representative strains, the authors showed that present populations are derived from 3 major Asian lineages, and that a diversification of effectors occurred within each of them. Some races tend to disappear across time while others become predominant (Quibod et al. 2016). A larger genomic analysis of 91 strains from the Philippines collected over 40 years was conducted to investigate the consequences of *Xa4* deployment on *Xoo* populations. Using phylogenetic-based genome-wide association between SNPs and phenotypic dataset, the study provides the evidence that pathogen adaptation to *Xa4* occurred in multiple ways (Quibod et al. 2020). In China, whole genome sequencing of 247 strains collected over 30 years highlighted six lineages with two of them regrouping 70% of the strains. The authors show that while *Xoo* population structure is globally shaped by their geographical origin and the subspecies of cultivated rice, there is also a rapid virulence dynamic determined by *R* genes selection pressure in the field (Zheng et al. 2019).

Multilocus Variable-Number Tandem-Repeat (VNTR) Analysis (MLVA) for the molecular typing of pathogens is a useful tool to investigate genetic diversity and population structure avoiding costly whole genome sequencing. VNTRs are DNA motifs repeated in differential number of copies in the genome of eukaryotic and prokaryotic species. Differences in the number of repetitions are essentially determined by recombination between repeats and by stepwise INDEL events caused by slip-strand mispairing. The latter results in the addition or deletion of one repeat and is considered the major mechanism of variation for VNTR. A stepwise mutation model is the usual method of analysis employed in MLVA and allows to robustly investigate neutral descendance relationships between strains. For surveillance and epidemiological studies of *X. oryzae* a MLVA scheme based on 16 loci (MLVA-16) was developed and used to successfully discriminate 186 strains from 12 countries including 59 from Africa (Poulin et al. 2015). While developed for large scale studies, this tool is also suitable to discriminate populations at a small geographical scale.

In West Africa, where BLB was described for the first time in Mali in 1979, the disease is widely present today (Verdier et al. 2012) causing yield losses from 20 to 80% (Sileshi and Gebeyehu 2021). African strains of *Xoo* form a genetic lineage distinct from Asian ones and differ by many traits (Gonzalez et al. 2007; Poulin et al. 2015). First, they contain a smaller number of TALEs, usually nine. Whole genome sequencing of 11 African strains including nine from Mali highlighted that two TALE groups are highly conserved between strains (TalE and TalC) while seven (TalG, TalD, TalH, TalI, TalF, TalB and TalA) display 2 to 6 RVDs polymorphisms and/or differences in their number of repeats (Doucouré et al. 2018; Tran et al. 2018a). Second, most African *Xoo* activate the susceptibility gene *ERF#123*, in addition to *SWEET14* (Doucouré et al. 2018; Tran et al. 2018a). Third, African *Xoo* form specific races, notably because they are controlled by *Xa1*, contrary to most Asian strains (Gonzalez et al. 2007; Ji et al. 2020). Nine races (named A1 to A9) were described in West Africa, two of which were reported in Burkina Faso (Tekete et al. 2020). Finally, African *Xoo* activate specific resistance QTLs and carry unique avirulence TALEs (Djedatin et al. 2016; Lachaux et al. 2022).

Successful strategies of *R* genes deployment rely on knowledge of the bacterial populations and their spatio-temporal variability. Thus, the aim of this study was to assess the diversity and the evolutionary dynamic of *Xoo* populations in Burkina Faso in order to improve their control. We describe the pathogenic and genetic characterization of a collection of 177 strains collected between 2003 and 2018 in three regions of Burkina Faso with a focus on strains collected from 2016 to 2018 in the locality of Bagré. Combining race pathotyping, MLVA analysis and TALE repertoires profiling, we show a high diversity at the scale of the country and the field and a highly dynamic evolution of some TALE. Variations in the occurrence of TALome patterns across years reveals a high potential for adaptation and brings knowledge that will be essential to anticipate resistance genes breakdown and to improve their durability.

## Results

### Constitution of a Large Collection of *Xoo* Strains from Burkina Faso

**Fig. 1:**
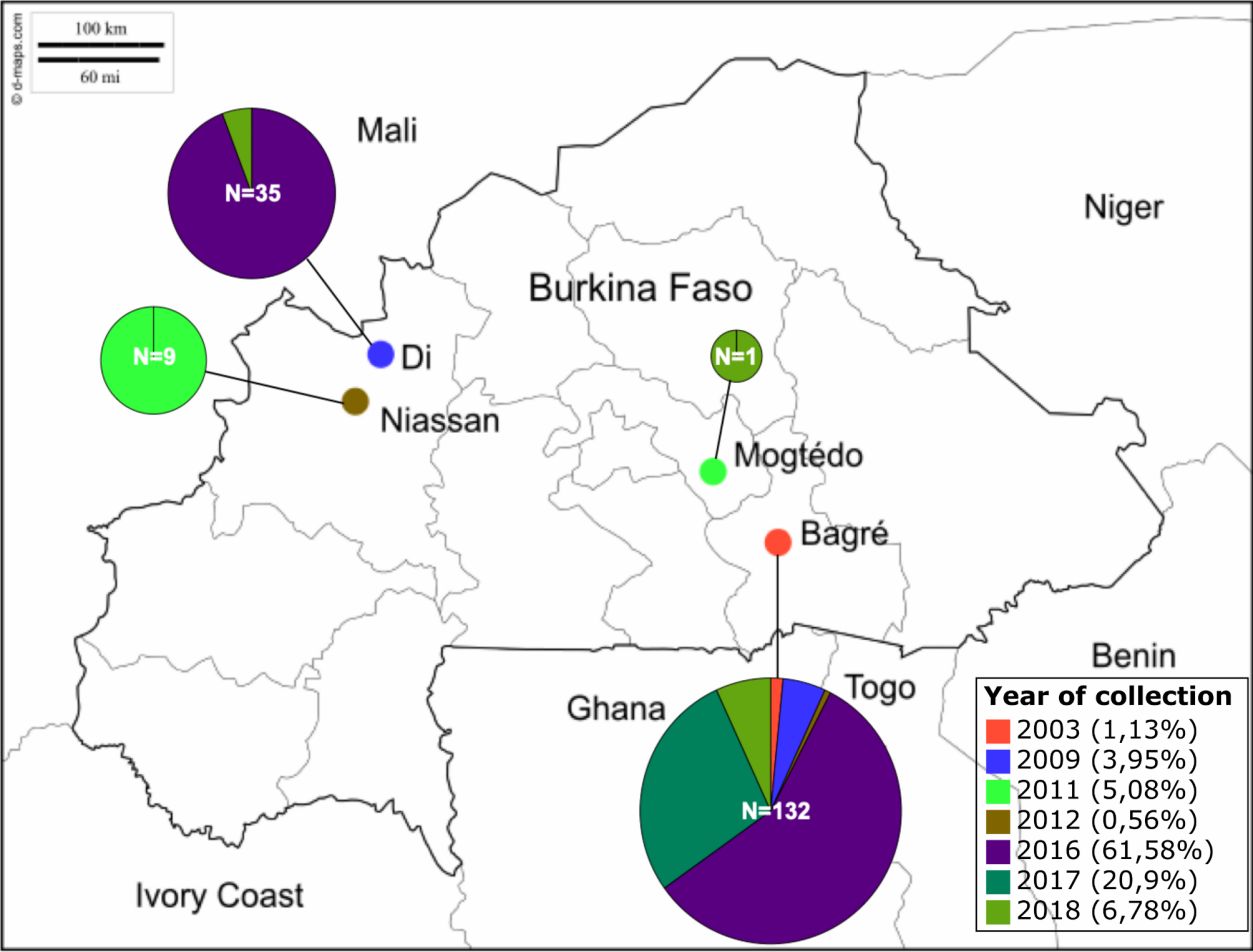
Distribution per site and per year of a collection of 177 strains of *Xanthomonas oryzae* pv. *oryzae* from Burkina Faso

In order to investigate the genetic structure of *Xoo* populations and the diversity of their TAL effector repertoires in Burkina Faso, a collection of 177 strains from two major rice producing areas was used (Table S1, Fig. 1). A total of 158 strains was isolated from leaves collected in 2016 (n = 109), 2017 (n = 37) and 2018 (n = 12) and added to 19 strains isolated between 2003 and 2012 (Table S1, Fig. 1). Species and pathovar of the isolated colonies were confirmed by leaf clipping inoculations and observation of the typical BLB symptoms 14 days after inoculation.

In the Boucle du Mouhoun region two localities were sampled: Di, in 2016 and 2018 where the incidence of the disease varied from 5 to 99.6%, and Niassan in 2011. In 2017 the incidence of the disease was low in Di and no *Xoo* could be isolated from the samples collected. In Di three different fields were prospected and 35 *Xoo* strains were isolated. In the Centre Est region, the locality of Bagré distant of 450 km from Di and the locality of Mogtédo were sampled. In Bagré where nine fields were prospected regularly between 2016 and 2018, the incidence of the disease varied from 10 to 86.5%. With the 10 strains collected in 2003, 2009 and 2012, a total of 132 strains from Bagré were isolated. In Mogtédo, a prospection was done only in 2018 and allowed to isolate only one *Xoo* strain.

### MLVA Genotyping of the Burkinabe Strains

The MLVA scheme designed by Poulin et al., based on 16 microsatellites loci was optimized after resequencing of each loci of the reference *Xoo* strain BAI3 from Burkina Faso (Poulin et al. 2015). Four VNTR loci, Xo_G09, Xo_G15, Xo_G80, which did not reveal any tandem-repeat (TR) variation, and Xo_83, are considered to be diagnostic markers of the African strains of *Xoo* (n = 59) (Poulin et al. 2015). Two loci were removed because they were not useful for epidemiological purpose, Xo_G58 which contains two different types of repeats and Xo_G88 for which no amplification was obtained on *Xoo* strain BAI3. Our collection of African strains (n = 177) confirmed the diagnostic value of VNTRs with Xo_G09, Xo_G15, Xo_G80 and Xo_G83 being monomorphic. The resulting scheme is constituted of 10 polymorphic loci (mix 1, 2 and 3) and 4 monomorphic loci (mix 4) in *Xoo* strains from Burkina Faso (Table S2).

The genotype accumulation curve shows that the diversity of the 177 strains of *Xoo* isolated in Burkina Faso is well covered by the MLVA-14 scheme (Fig. S1), allowing to discriminate 22 haplotypes (Table 1). The genotypic diversity of *Xoo* from the Center East region is greater than from the Boucle du Mouhoun, as estimated by the richness based on rarefaction procedure with eMLG values of 12.78 and 4.0, respectively. The Simpson index of genotypic diversity was slightly greater in the Center East region while the index of Nei’s gene diversity was greater in the Boucle du Mouhoun region with values of 0.870 and 0.161 against 0.668 and 0.324, respectively (Table 1). The genotypic diversity and genetic diversity of the whole collection were 0.901 and 0.310, respectively.

**Table 1:** Global genetic diversity estimated from MLVA-14 data of *X. oryzae* pv. *oryzae* (n = 177) for the 2 sampled region collections

		Genotypic Richness			Genotypic diversity		
Region	N^a^	MLG^b^	eMLG^c^	SE^d^	H^e^	Simpson index	Hexp^f^
Boucle du Mouhoun	44	4	4	0	1.1920	0.66838	0.324297
Center East	133	19	12,7822	1,5131	2.351316	0.87037	0.161239
Total	177	22	13,8611	1.6055	2.55343	0.90114	0.309950

^a^ Number of samples ^b^ Number of haplotypes ^c^ Expected MLG based on rarefaction

^d^ Standard error from rarefaction ^e^ Shannon-Wiener Index ^f^ Nei’s unbiased gene diversity

**Fig. 2:**
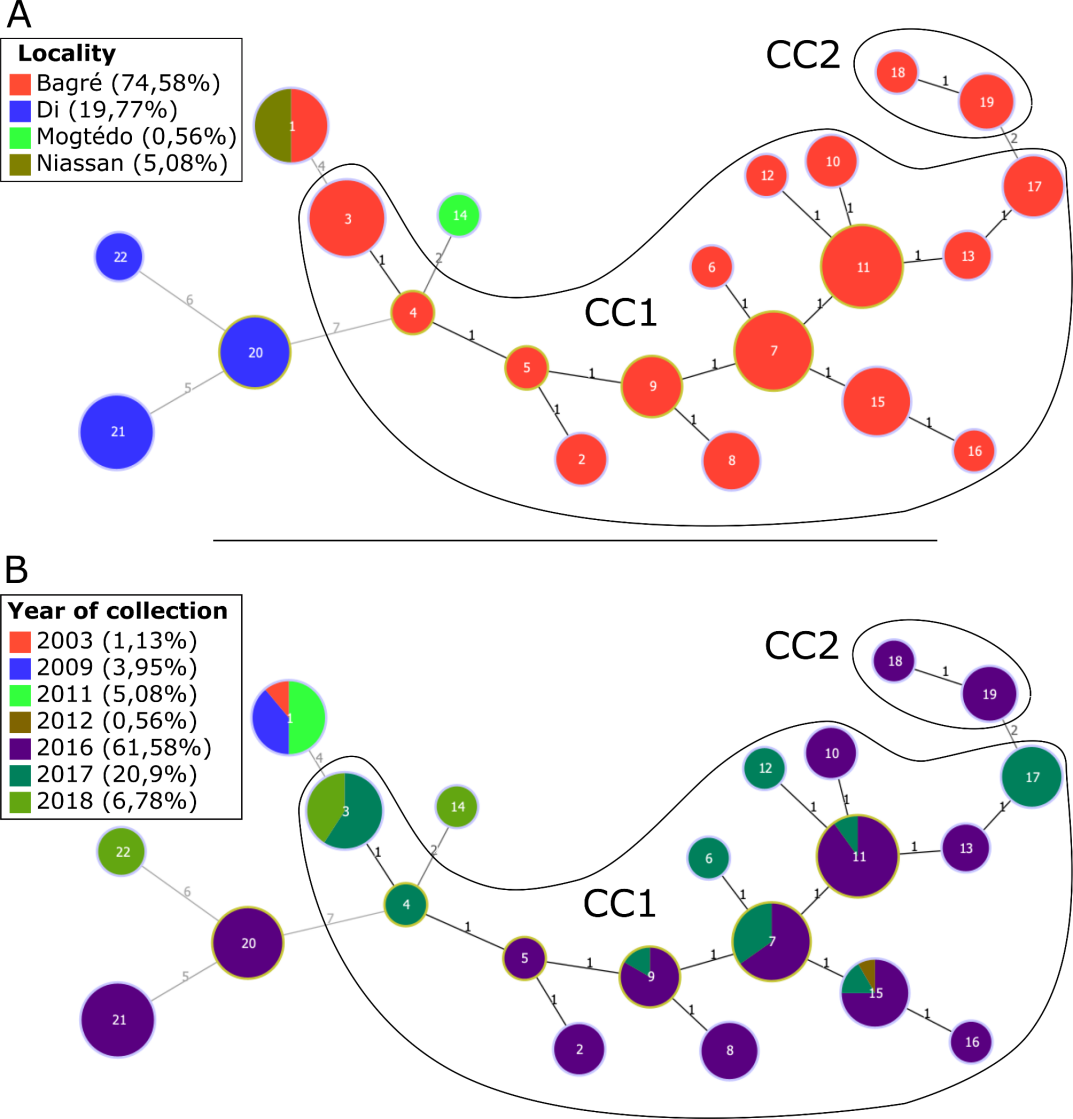
Minimum spanning tree of *Xanthomonas oryzae* pv. *oryzae* populations in Burkina Faso by (**A**) Locations and (**B**) Years of collection Each haplotype is represented by a circle whose size is correlated to the number of strains it contains. The haplotype number is indicated inside the circles. The number of different loci (distance) between two haplotypes is indicated between the linked haplotypes. Clonal Complex (CC) defined as groups containing only single locus variants are circled in black

**Fig. 3:**
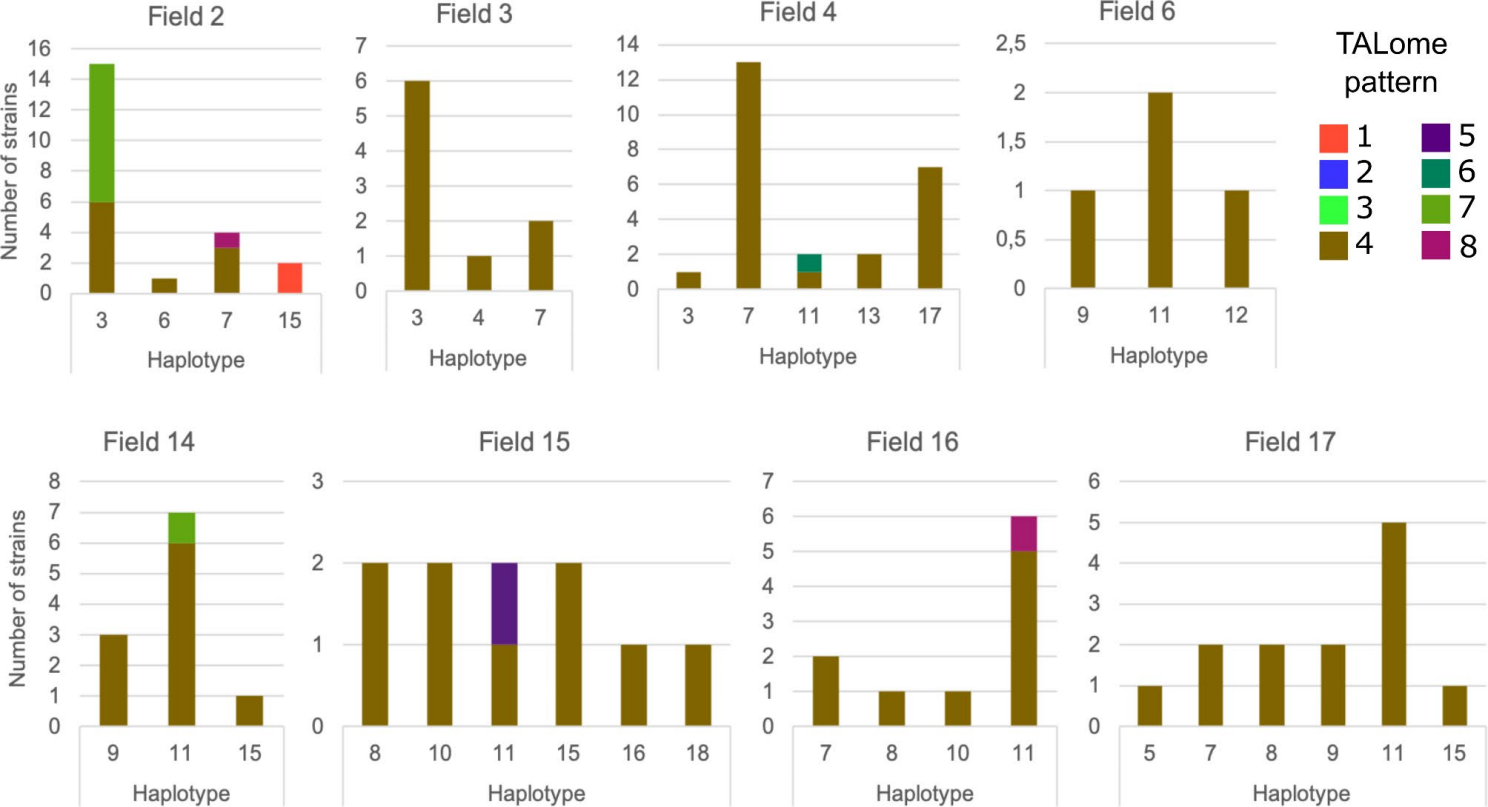
Distribution of the 8 TALome patterns within haplotypes present in 8 fields in Bagré

A minimum spanning tree was built to investigate the relationships between haplotypes and allowed to identify two clonal complexes (CCs) defined as groups of single locus variants (SLV), i.e. that share 13 out of 14 alleles, and five singletons which are haplotypes differing from any other for more than one locus (Fig. 2). All haplotypes originated from a single locality, except haplotype #1 which is shared by strains collected from two localities before 2012 (Fig. 2A). The two CCs are composed only by strains from Bagré, with the large CC1 containing 15 haplotypes (n = 118), and CC2 containing only two haplotypes (n = 5). The two CCs were separated by only a double-locus variation. The primary founder of this clonal complex, defined as the haplotype with the highest number of SLVs, could be haplotypes 7 (5 SLVs and 9 DLVs) or 11 (4 SLVs and 8 DLVs). The five singletons grouped all the strains isolated from the three other localities. The nine strains from Niassan shared the same haplotype #1, which differs from CC1 by at least four VNTR loci. This haplotype #1 is also shared by the strains from Bagré isolated in 2003 and 2009. The unique strain from Mogtédo is a double-locus variant of strains from Bagré. The strains from Di were of three haplotypes differing from each other and from the other haplotypes by at least 5 loci. In this locality each haplotype is associated specifically to a field of collection, suggesting independent introduction events in the 3 sampled fields. On the contrary, all the strains from Bagré isolated from a given field were divided in at least three haplotypes (Fig. 3). Pairwise comparisons of the 3 localities (Mogtédo being ignored because represented by only one strain) show a highly significant R_ST_ indicating a strong genetic differentiation between them (Table 2). MLVA haplotypes that are genetically distant from each other were isolated either at geographically distant sites or more than four years apart.

**Table 2:** Genetic differentiation of *Xanthomonas oryzae* pv. *oryzae* between sites and between year in Bagre locality

	Di	Bagre	Niassan		2017	2016	2018
Di	0.00000			2017	0.00000		
Bagre	0.77598	0.00000		2016	0.36057	0.00000	
Niassan	0.69387	0.64791	0.00000	2018	0.44511	0.89982	0.00000

Significance level of R_ST_ pairwise comparisons: P < 0.001

The majority of the haplotypes is composed by strains collected in the same year or in two consecutive years (Fig. 2B). Only two haplotypes grouped strains that were isolated more than two years apart (haplotypes #1 and #7). Pairwise comparison of populations collected in Bagré in 2016, 2017 and 2018 estimated by R_ST_ showed a significant genetic differentiation between these 3 years (Table 2). All strains collected before 2012 belong to the same haplotype.

### Races of *Xoo* Present in Burkina Faso and Characterization of TALE Repertoires

Previous studies carried out in Burkina Faso described the presence of two races, race A1 and A2, while seven other races including the race A3 were found in the neighboring country Mali (Gonzalez et al. 2007; Tekete et al. 2020).

Thirty-seven strains of *Xoo* representing the diversity of the collection were inoculated on the parental line IR24 and its near isogenic lines IRBB3, IRBB4, IRBB5 and IRBB7 which allow to discriminate races among West African strains of *Xoo*. Fourteen days post inoculation plants with lesion lengths inferior to 10 cm were considered as resistant (Table 3). Interestingly, all the strains collected between 2003 and 2009 and assessed in this study belong to a new race (A10) characterized by its avirulence on IRBB4 and virulence on IRBB3. The reference Burkinabe strain BAI3 which was previously classified as race A1, also belongs to race A10. On the other hand, all the strains tested and collected since 2016 belong to race A3.

**Table 3:** Race characterization of 37 strains of *Xoo* from Burkina Faso

	Isogenic lines						
Strain	IR24	IRBB3	IRBB4	IRBB5	IRBB7	RACE	Source
BAI1	S	S	MR	R	S	**A1**	Gonzalez et al. 2007
BAI2	S	S	MS	R	S	**A1**	Gonzalez et al. 2007
BAI3	S	S	R	R	S	**A10**	This study
BAI4	MS	R	R	R	R	**A2**	Gonzalez et al. 2007
BAI28	S	S	MR	R	S	**A10**	This study
BAI33	S	MS	MR	R	S	**A10**	This study
BAI50	S	MS	MR	R	MS	**A10**	This study
BAI55	S	MS	MR	R	S	**A10**	This study
BAI111	R	R	R	R	R	**A3**	This study
BAI145	R	R	R	R	R	**A3**	This study
BAI146	R	R	R	R	R	**A3**	This study
BAI166	R	R	R	R	R	**A3**	This study
BAI167	R	R	R	R	R	**A3**	This study
BAI168	R	R	R	R	R	**A3**	This study
BAI169	R	R	R	R	R	**A3**	This study
BAI170	R	R	R	R	R	**A3**	This study
BAI181	R	R	R	R	R	**A3**	This study
BAI182	R	R	R	R	R	**A3**	This study
BAI183	R	R	R	R	R	**A3**	This study
BAI192	R	R	R	R	R	**A3**	This study
BAI196	R	R	R	R	R	**A3**	This study
BAI197	R	R	R	R	R	**A3**	This study
BAI202	R	R	R	R	R	**A3**	This study
BAI204	R	R	R	R	R	**A3**	This study
BAI220	R	R	R	R	R	**A3**	This study
BAI221	R	R	R	R	R	**A3**	This study
BAI222	R	R	R	R	R	**A3**	This study
BAI235	R	R	R	R	R	**A3**	This study
BAI236	R	R	R	R	R	**A3**	This study
BAI237	R	R	R	R	R	**A3**	This study
BAI250	R	R	R	R	R	**A3**	This study
BAI255	R	R	R	R	R	**A3**	This study
BAI257	R	R	R	R	R	**A3**	This study
B2E15	R	R	R	R	R	**A3**	This study
B2E16	R	R	R	R	R	**A3**	This study
B3E3	R	R	R	R	R	**A3**	This study
B6E2	R	R	R	R	R	**A3**	This study
M3-4	R	R	R	R	R	**A3**	This study
BAI216	R	R	R	R	R	**A3**	This study
BAI189	R	R	R	R	R	**A3**	This study

**Fig. 4:**
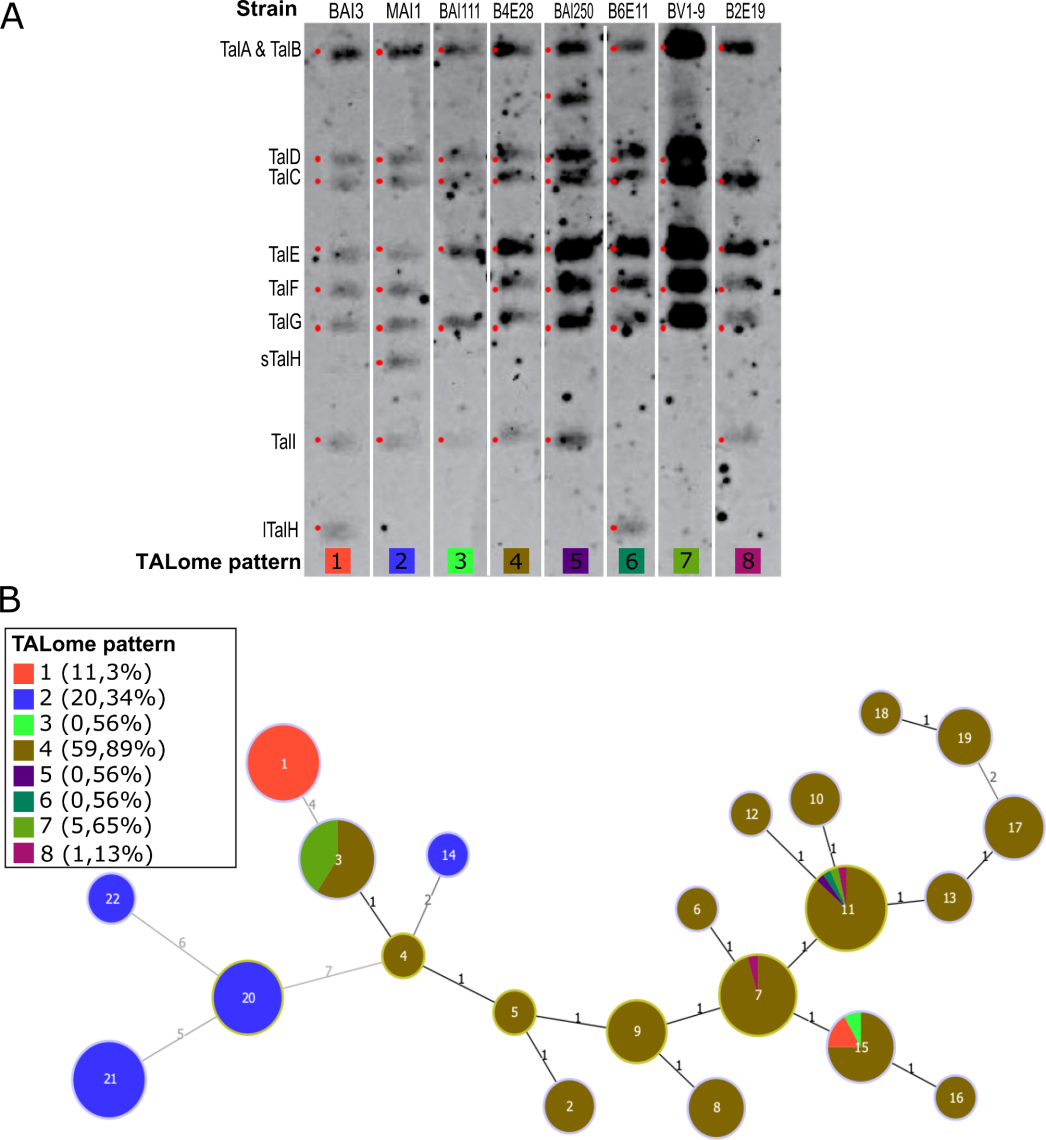
Diversity and distribution of TALome patterns across haplotypes of *Xanthomonas oryzae* pv. *oryzae* in Burkina Faso **A**. The 8 TALome patterns identified in the 177 strains of *Xoo* assessed and revealed by RFLP. Total genomic DNA of each strain was digested with the enzyme BamH1-HF, which cuts on either side of the central region of TALE repeats **B**. Projection of TALome patterns of each strain on the minimum spanning tree of the populations of *Xoo* in Burkina Faso. Each haplotype is represented by a circle whose size is correlated to the number of strains it contains. The haplotype number is indicated inside the circles. The number of different loci (distance) between each haplotype is indicated between the linked hapolypes

Nine TALE groups were defined for African *Xoo* strains from TalA to TalI, and several studies have shown that their size, sequence and sometimes presence vary across strains (Doucouré et al. 2018; Tran et al. 2018b). In order to have an overview of the diversity of *Xoo* TALomes in Burkina Faso, Restriction Fragment Length Polymorphism (RFLP) experiments were performed. Among the 177 strains assessed, we were able to distinguish 8 different patterns (Fig. 4A). The sequences of TALEs of BAI3 and MAI1 being available (Tran et al. 2018a), *tale* names were attributed to bands according to their size in these two strains. In BAI3 and MAI1, four TALEs have the same size but hold 2 to 6 RVDs polymorphisms that cannot be detected in this analysis. One of them, TalH, is known to vary in size so that “lTalH” and “sTalH” refer to the large and short versions of TalH, respectively. TALEs in other groups are more conserved in terms of number of repeats and RVD sequences. As previously reported, BAI3 (TALome pattern 1) contains sTalH and MAI1 (TALome pattern 2) has the larger lTalH with 4 more repeats (Fig. 4A). Six new TALome patterns are reported here. The TALome patterns 3, 4, 5, 7 and 8 do not have bands corresponding to any of the *talH* genes. TALome pattern 4 has no talH and is shared by 60% of the strains. TALome pattern 3, which was found in only one strain, lacks *talF* and *talH* bands. In TALome pattern 5 a so far unknown band which is slightly smaller than *talA/talB* bands is detected. It may result of a recombination of the *talH* gene. This pattern was found in only one strain. The *talI* band is absent from 6,2% of the strains (TALome patterns 6 and 7). Finally, the TALome pattern 8 (two strains) does not contain bands corresponding to *talH* and *talD*.

### Distribution of the TALome Patterns Across 177 Strains of *Xoo* from Burkina Faso

In order to analyze how TALomes evolve in the field, TALome patterns were projected on the minimum spanning tree based on MLVA data (Fig. 4B). Haplotypes 1, 3, 7, 11 and 15 contain strains corresponding to several distinct TALome patterns, which suggests a recent evolution of the TALEs repertoire of these strains. All the strains collected between 2003 and 2009 originating from two distant localities share TALome pattern 1, which was only found in two haplotypes. TALome pattern 2 is found in 4 haplotypes and represents the only TALome pattern found in Di and Mogtédo.

Finally, the diversity of haplotypes and TALome patterns within 8 fields was analyzed in details in Bagré. In general, several haplotypes were found in each field, and most of the time the haplotypes were closely genetically related and grouped within clonal complexes (Fig. 3). Interestingly, in 5 fields (Fields 2, 4, 14, 15 and 16) we were able to detect up to 4 different TALome patterns. The presence of different TALomes within a same haplotype across years suggests that pattern 4 could be at the origin of the pattern 7 characterized by the apparent extinction of the TalI group in the haplotype 3 between 2017 and 2018. The presence of both patterns in 2016 in the haplotype 11 corroborates this hypothesis (Fig. S2). TALome patterns 5, 7 and 8 were found in the haplotype 11 in 2016 but were not found in this haplotype and its SLVs in 2017 and 2018. It could be that the sampling was less exhaustive in 2017 and 2018 or that these TALomes did not confer a selective advantage favorizing their maintenance across years. Monitoring the appearance or maintenance of TALome patterns in the coming years will allow to evaluate which combination of TALEs confers a selective advantage.

## Discussion

In this study we aimed at describing the population structure of *Xoo* in Burkina Faso through the analysis of the diversity of neutral (VNTR loci, which are supposed to evolve mostly by slip-strand mispairing and are not considered to be under selection) and virulence markers (TALEs, that are highly evolvable and probably under selection). For this purpose, we characterized the genetic and pathotypic diversity of a collection of 177 *Xoo* strains using MLVA, TALome and race profiling. Our data show that populations are structured according to geographical origin and year of collection, and that different TALome patterns can be found in distinct strains originating from a same field, or even the same MLVA haplotype, allowing to build scenarios about the evolutionary dynamics of *Xoo* populations in Burkina Faso.

Numerous studies have shown the potential of microsatellites to analyze pathogen population structure and epidemiology at local to regional scales for different *Xanthomonas* species (López-Soriano et al. 2016; Pruvost et al. 2019; Vernière et al. 2014; Vancheva et al. 2021). As an example, a MLVA scheme allowed to discriminate *Xanthomonas citri* pv. *citri* lineages inside countries or groups of neighboring countries better than the AFLP method (Bui Thi Ngoc et al. 2009). The MLVA-14 scheme used in this study was adapted from an MLVA-16 scheme developed previously to analyze a world collection of *X. oryzae* pvs. *oryzicola* and *oryzae* with 186 strains of *Xoo* including 59 from African countries (Poulin et al. 2015).

Although the MLVA-16 scheme was designed for regional level studies, this scheme showed a potential for discriminating strains at smaller geographical and time scales. Here, we applied this scheme to study the *Xoo* population structure at the locality and field level, supported by an extensive sampling over 2016 to 2018 in the irrigated perimeter of the Bagré locality. We were able to discriminate strains at the field level and monitor genetic variation occurring within a short temporal scale.

### Genetically Distant MLVA Haplotypes are Separated Geographically or in Time

Genetically distant MLVA haplotypes were either isolated at geographically distant sites or more than four years apart. For strains isolated from 2016 to 2018 in Bagré, a clear clustering could be observed. Overall no haplotype grouped strains from different localities, i.e. Bagré, Mogtedo and Di, suggesting a limited exchange of plant material between distant localities. A similar scenario was observed in China based on the core SNPs analysis of 237 whole genome sequences of *Xoo* strains, showing that isolates genetic structuration correlates with their geographical origin (Zheng et al., 2019).

In contrast, a study on populations of *Xanthomonas phaseoli* pv. *manihotis* in Colombia revealed no genetic differentiation between strains from different localities, in agreement with previous reports about the exchange of cassava cuttings between ecozones (Rache et al. 2019; Trujillo et al. 2014).

### Two Contrasted Local Epidemiological Situations Highlighted in the Localities of Di and Bagré

Each of the three different fields sampled in Di contained a unique and genetically distant haplotype (Fig. 2). In contrast strains isolated from the nine fields sampled in Bagré were more diverse but closely related, with a major clonal complex closely related to a smaller one. Among the eight MLVA haplotypes detected in Bagré and represented by more than four strains, seven were distributed in several fields. Three SLV haplotypes, i.e. #7, #11 and #15 were distributed in five or seven fields out of nine sampled in Bagré. This may reflect an intense epidemiological dynamic, which could be the result of exchange of infected material such as seeds, or natural dispersion of bacterial strains as these fields are very close geographically (Fig S1). In contrast, our results in Di do not support such scenario, with a reduced disease incidence, little variability and no apparent exchange of inoculum between fields. Further, the relatively high genetic distance between the three field populations of Di suggest independent introduction events of the disease. Di is localized at the border between Burkina Faso and Mali and is the place of intense uncontrolled exchanges of agricultural products across both countries. Complementary analysis including *Xoo* strains from Mali would be required to investigate if there might have been exchange of strains between the two countries. The reasons why the two locations present contrasted epidemiological situations remain to be assessed; it could be simply due to different agricultural, but differences in strains aggressiveness or in the rice cultivars might also be implied.

### MLVA Haplotypes Survive from One Year to the Next and Evolve as Clonal Complexes in Bagre

Most haplotypes contained strains collected the same year or two consecutive ones, and two haplotypes grouped strains that were isolated more than two years apart (#1 and #15). These results can be explained by seed transmission of *Xoo* and the absence of new introductions. It could also suggest that *Xoo* is maintained in the field, in crop residues or in weeds that persist during the off-season. The fact that strains collected over 3 years from the same location are in the same CC corroborates the hypothesis of little external inoculum, but some survival from one season to the next through seed or other reservoirs, and a dynamic evolutionary context resulting in bacterial diversification from a single or a few founders. Such basic epidemiological information is essential for prophylactic measures against the development of BLB epidemics. Sampling wild species at the edges of the field and crop residues between seasons (Lang et al. 2019), as well as characterizing the role of seeds in disease transmission will help assess their relative importance in the epidemiology of the disease.

### Emergence and Establishment of the Race A3 in Burkina Faso

The first characterization of *Xoo* races in Africa was conducted with 21 strains including 4 from Burkina Faso that were collected in 2003 (Gonzalez et al. 2007). At that date races A1 and A2 were the only one found in Burkina Faso while race A3 was exclusively found in Mali.

Recently, six new races, A4 to A9, were reported in Mali, reflecting an important diversification during the last decade (although this might be the result of limited number of isolates before 2007) (Tekete et al. 2020). By profiling the race structure of 37 strains from Burkina Faso collected between 2003 and 2018, we observe a totally different situation in Burkina Faso. Indeed, if a new race (A10) was identified among the strains collected in 2011 and before, it appears that the strains collected after 2012 and from 4 different localities all belong to race A3. The apparent extinction of races A1, A2 and A10 is probably not due to a bias in the sampling as strains from several localities including the one of the old strains were analyzed. It is more probable that race A3 benefits from a selective advantage towards rice varieties cultivated in Burkina Faso. This phenomenon was evidenced in the Philippines where the large deployment of the *R* gene *Xa4* resulted in the expansion of races able to overcome this resistance (Quibod et al. 2020). Curiously, race A3 is avirulent on IR24, the parental variety of the NILs, which would carry the *R* gene *Xa18*. The cognate *Avr* gene is not identified yet but it could be a virulence factor which would confer a selective advantage to race A3 as is the case for AvrXa7 which contributes to susceptibility by inducing the *S* gene *SWEET14* but elicits resistance in plants carrying *Xa7* (Antony et al. 2010; Chen et al. 2021). No relation between races and TALomes patterns was observed in our study, which is not surprising as the NILs containing *R* genes are not known to respond to African TAL effectors. Further studies of the varieties cultivated in Burkina Faso will be necessary to understand why the race A3 became predominant.

### An Important Diversity of TALome Patterns in Burkina Faso

Our analysis revealed the presence of 8 different TALome patterns within the Burkinabe *Xoo* population. Patterns #1 and #2 were already described for strains BAI3 and MAI1, respectively (Gonzalez et al. 2007). Whole genome sequencing of 11 African *Xoo* strains including 9 from Mali and 1 from Burkina Faso revealed the existence of 9 TALE paralogs named TalA to TalI (Doucouré et al. 2018; Tran et al. 2018a). Two of them, TalC, which is a major virulence factor, and TalE, which has no known function, are strictly conserved across strains. The 7 others present two to six polymorphisms in their RVDs. However, RFLP analysis of a collection of Malian strains of *Xoo* revealed only 4 different TALome patterns, three of which were represented by single strains, and the fourth containing all the other strains analyzed (Doucouré et al. 2018). Among the 8 TALome patterns identified in Burkina Faso, 4 are preponderant with 13 to 116 representative strains, and 4 are rare with two TALome patterns represented by two strains and two represented by only one strain. These results highlight an important *tal* gene diversity of the *Xoo* population from Burkina Faso. In our study, 5 TALE groups among the 9 previously identified varies by their presence/absence and interestingly, they all belong to groups for which polymorphisms in RVDs sequence were already observed.

### Scenario for TALome Evolution in Burkina Faso

With the development of long-read sequencing and, consequently, accurate assembly of repetitive sequences, more and more TALE sequences have become available allowing comparative studies in order to understand the mechanisms driving their evolution (Perez-Quintero and Szurek 2019). Several mechanisms have been proposed for the evolution of TALEs including point mutations by substitutions (Erkes et al. 2017), recombination between repeat domains of different TALEs as exampled in the TalF group of the african *Xoo* strains BAI3 and MAI1 (Tran et al. 2018a), deletion or duplication of one or several repeats as examplified in the TalH group variants in Malian *Xoo* (Doucouré et al. 2018) and finally *tal* gene duplications or loss. Analysis of TALome patterns distribution within MLVA haplotypes revealed that 5 of them (#1, #3, #7, #11 and #15) contain several patterns, suggesting a recent evolution of their TALEs repertoire. Particularly TalI and TalH seems to be under evolution. TALome patterns 4 and 7 which are each found in 2 different haplotypes differ from each other in the presence/absence of *talI*. In one haplotype the 2 TALome patterns belong to strains collected the same year, and in the other one they correspond to strains collected during two consecutive years. Whole genome sequencing of the 4 strains representing the 2 TALome patterns in each haplotype will be necessary to investigate the mechanism which has caused the disappearance of *talI*.

In some case recombination can lead to new functional effectors as examplified with TalF from the strain MAI1, which contains 5 repeats identical to the one of TalG and contrary to TalF from strain BAI3, is able to induce the *S* gene *OsSWEET14* (Tran et al. 2018a). Mutation in or deletion of a *tale* gene could also prevent the elicitation of a resistance gene. It is tempting to speculate that the independent loss of *talI* and *talH* in several TALome patterns could be the result of a selective pressure exerted by a resistance gene deployed in the rice varieties grown in Bagre. To confirm this, sequencing, further sampling, and knowledge about the genetic background of the cultivated varieties there will be required. Sampling carried out in 2019–2021 should allow to establish whether this TALome pattern becomes dominant and whether the loss of *talI* confers an adaptative advantage.

Analysis of the variation of TALome patterns in a field where, supposedly, only one variety is grown, showed that for 5 of them, up to 4 different TALome patterns are present. This could evidence different ways of adaptation to the presence of one or more resistance genes as shown for the adaptation of Asian *Xoo* to the large deployment of the *R* gene *Xa4* (Quibod et al. 2020). It could also be the results of infections by different *Xoo* genotypes present in the environment. Moreover, it was shown that not only *R* genes can shape population but also environmental factors and farming practices which should also be considered in future surveys (Zheng et al. 2019).

## Materials and Methods

### Bacterial Strains, Media, Growth Conditions

Bacterial strains used in this study were all *Xanthomonas oryzae* pv. *oryzae* and are listed in table S1. Strains were cultivated for 48 h at 28 °C on PSA medium (10 g of peptone, 10 g of sucrose, 1 g of glutamic acid, 16 g of agar per liter of H_2_O).

### Collection of Strains

*Xoo* strains are from different origins and collections (table S1). Strains from leaves collected before 2012 were already published (Gonzalez et al. 2007; Poulin et al. 2015). Surveys for rice BLB disease were performed between September and October in 2016, 2017 and 2018 in major rice production areas of Burkina Faso. Leaves were collected according to the presence of typical BLB symptoms from *Oryza sativa* varieties. Bacterial isolations were performed as previously described (Adhikari et al. 1994). Rice leaves were ground and resuspended in 1mL of sterile water. 100µL was streaked onto plates of PSA medium containing cycloheximide (50 mg/L), cephalexin (40 mg/L) and kasugamycin (20 mg/L). Plates were incubated for 2 to 5 days at 28 °C. One colony per leave was then plated and inoculated on susceptible rice line in order to confirm their ability to produce BLB symptoms. Validated isolates were then conserved in glycerol (15%) at -80 °C.

### Pathogenicity Assays

Experiments were performed in rice fields in Burkina Faso in 2017 and 2019 and under greenhouse conditions at 26 °C and 80% relative humidity in 2018, 2019 and 2020. The parental rice line IR24 and its derived near isogenic lines IRBB3, IRBB4, IRBB5 and IRBB7 containing respectively the resistance gens *Xa3*, *Xa4*, *Xa5* and *Xa7* were used to determine races. The highly susceptible variety Azucena was used as control for disease ability of each strain. Leaves from 4- to 6-week-old plants were clipped with a bacterial suspension resuspended at an optical density at 600 nm (OD_600_) of 0.2 as previously described (Kauffman 1973). Symptoms were scored by measurement of lesion lengths 14 days post-inoculation. At least 8 leaves per strain were clipped for each experiment. Each strain has been tested at least 3 independent times and at least one time under field condition.

### Genotyping

In total, 177 strains were genotyped including the strain BAI3 collected in 2004 in Burkina Faso for which whole genome sequence is publicly available. The 16 loci described in Poulin et al were amplified and sequenced in order to validate the congruence between whole genome sequencing data and genotyping one. Each strain was plated on PSA and incubated at 28°C for 5 days in order to obtain single colonies. A loop of one isolated colony was then resuspended in 100uL of sterile water and lyzed at 95°C for 10mn. The multiplex PCR was realized with 1µl of the lysate using the QUIAGEN^®^ Multiplex PCR kit (Quiagen, Courtaboeuf, France) as described in (Poulin et al., 2015). Sixteen VNTR loci were amplified in a quadruplex PCR containing four primers labeled on their 5’ extremities with 6-FAM, NED, PET and VIC fluorescent dyes (Applied Biosystems). Conditions were optimized and described in table S3. 1µL of diluted amplicons was mixed with 0.3 µl of the GeneScan 600 Liz internal size standard for the first mix and the GeneScan 500 LIZ for the 3 other mix (Applied Biosystems). Capillary electrophoresis was performed using an ABI 3500 XL sequencer at the GenSeq Platform (University of Montpellier, France).

Amplicon sizes were scored with GeneMapper 4.0 software (Applied Biosystems) and then converted to a number of tandem repeats as described by Poulin et al., for all loci except G88 and G58 which were removed of the analysis.

### MLVA Analysis

The R package poppr v.2.8.6 (Kamvar et al. 2015), via the shiny interface (https://bioinfo-shiny.ird.fr/ShinyGenotyping) developed at IRD, was used to calculate the genotypic richness and diversity as well as the unbiased genetic diversity of Nei. PHILOViZ 2.0 version 2016 software was used to reconstruct phylogenetic relationships between different allelic profiles (haplotypes), which are represented by minimum spanning trees constructed using an algorithm combining the global optimal eBURST (goeBURST) and Euclidean distances (Francisco et al. 2012). Haplotypes differing by a single locus (SLV, single locus variant) were grouped into clonal complexes (CC). The genetic differentiation between group (R_ST_) was calculated with Arlequin v.3.5.2.2 (Excoffier and Lischer 2010). Allelic richness and private allelic richness were calculated using a rarefaction method implemented in HP-rare (Kalinowski 2005).

### RFLP Analysis

Genomic DNA was extracted using the Wizard Genomic DNA Purification kit (Promega, Charbonnières, France) following the manufacturer’s instructions. For each strain, 4 µg of total DNA was digested overnight with *BamH1*-HF (New England Biolabs Inc., Saint Quentin, France). Standard procedures for Southern blots were utilized (Ausubel et al. 1988). Digested DNA was resolved by electrophoresis in 1% agarose gels in Tris-Borate EDTA buffer at 50V for 72 h. Fragments were transferred in alkaline solution onto a nylon membrane (Roche) overnight and fixed by UV-crosslinking. The kit « DIG High Prime DNA Labelling and Detection Starter » (Roche) was used to revealed the TALome following manufacturer instructions. First, the membrane was incubated with agitation twice for 5 minutes in a 2X SSC solution (500 ml) at room temperature. Then, it is incubated under agitation successively in 75 ml of a “DIG Easy Granules” pre-hybridization solution for 30 min at 40°C, then in 75 ml of a “DIG Easy Granules” hybridization solution containing 10 µl of probe (25 ng/ml) overnight at 40°C. The probe used contains the coding sequence of the C-terminal *talA* region of BAI3 and was amplified by PCR using the GoTaq DNA Polymerase kit (Promega®) and the primers Tal-Ct_Fw2 (5’ GCGTTGGCCGCGTTGACCAA) et Tal-Ct_Rv2 (5’ GGGGCCGCATCTTGTTCCCA) (Yu et al. 2011). The Wizard SV Gel and PCR clean-Up System kit (Promega®) was used to purify the Southern Blot probe.

## Electronic Supplementary Material

Below is the link to the electronic supplementary material.

**Additional file 1: Table S1**. Strains analyzed in this study and metadata

**Additional file 2: Table S2**. MLVA scheme used and number of alleles at each locus

**Additional file 3: Table S3**. PCR condition and dilution used for genotyping

**Additional file: Fig. S1** Rarefaction curve of genotypes obtained with the MLVA-14 scheme on the 177 Xoo strains from Burkina Faso. The four monomorphic loci were removed of this analysis

**Additional file: Fig. S2** Distribution of TALome profiles in haplotypes containing Xoo strains collected in Bagr? between 2016 and 2018

## Data Availability

The datasets supporting the conclusions of this article are provided within the article and its supplementary information files.
