# Supplementary figures and images for: Genetic Structure and TALome Analysis Highlight a High Level of Diversity in Burkinabe Xanthomonas Oryzae pv. oryzae Populations
Source: Rice (N Y). 2023 Jul 31;16:33. doi: 10.1186/s12284-023-00648-x (PMC10390441; doi:10.1186/s12284-023-00648-x)

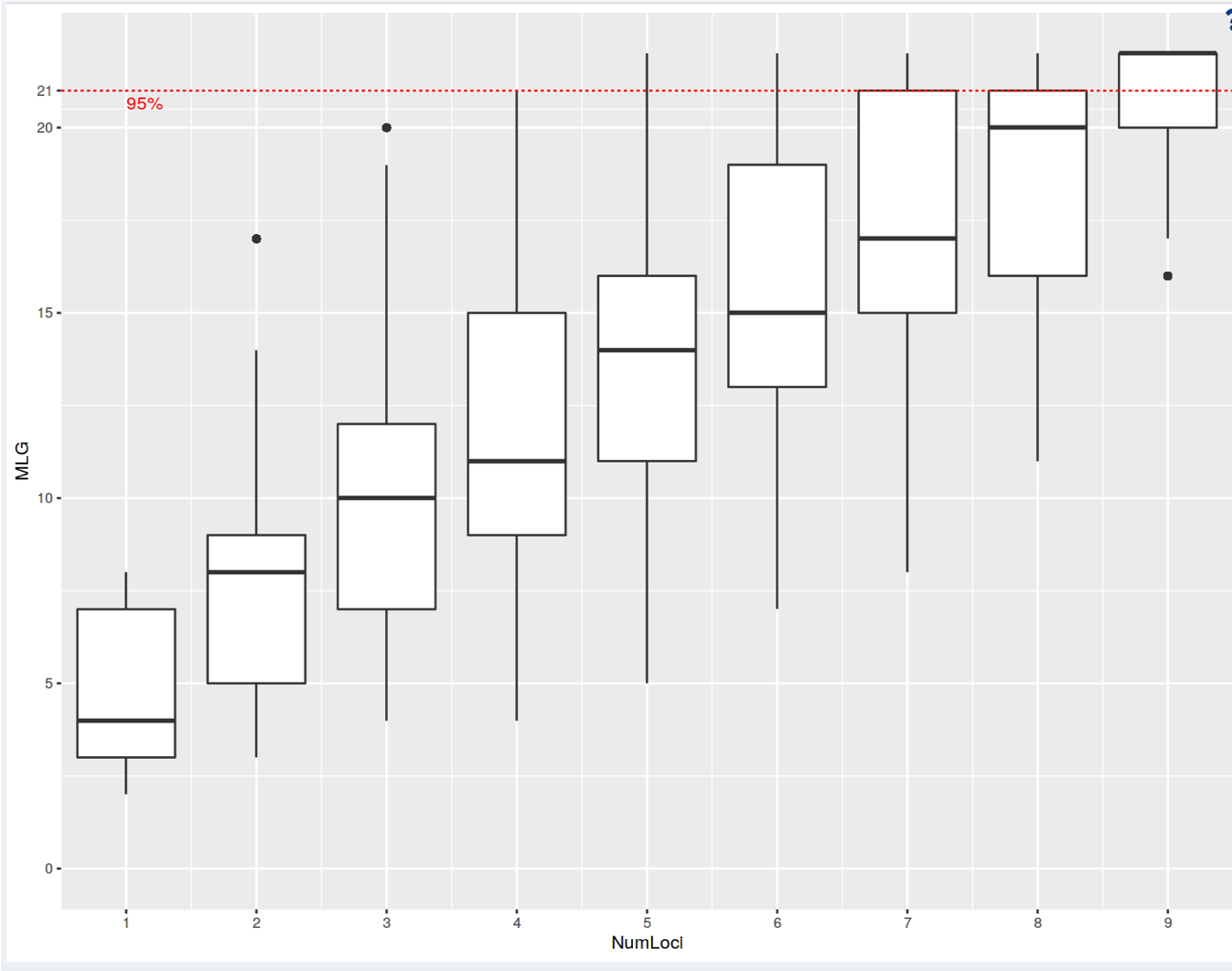

Supplement: Supplementary file 1 — Additional file 1: Table S1. Strains analyzed in this study and metadata [file 12284_2023_648_MOESM1_ESM.png]

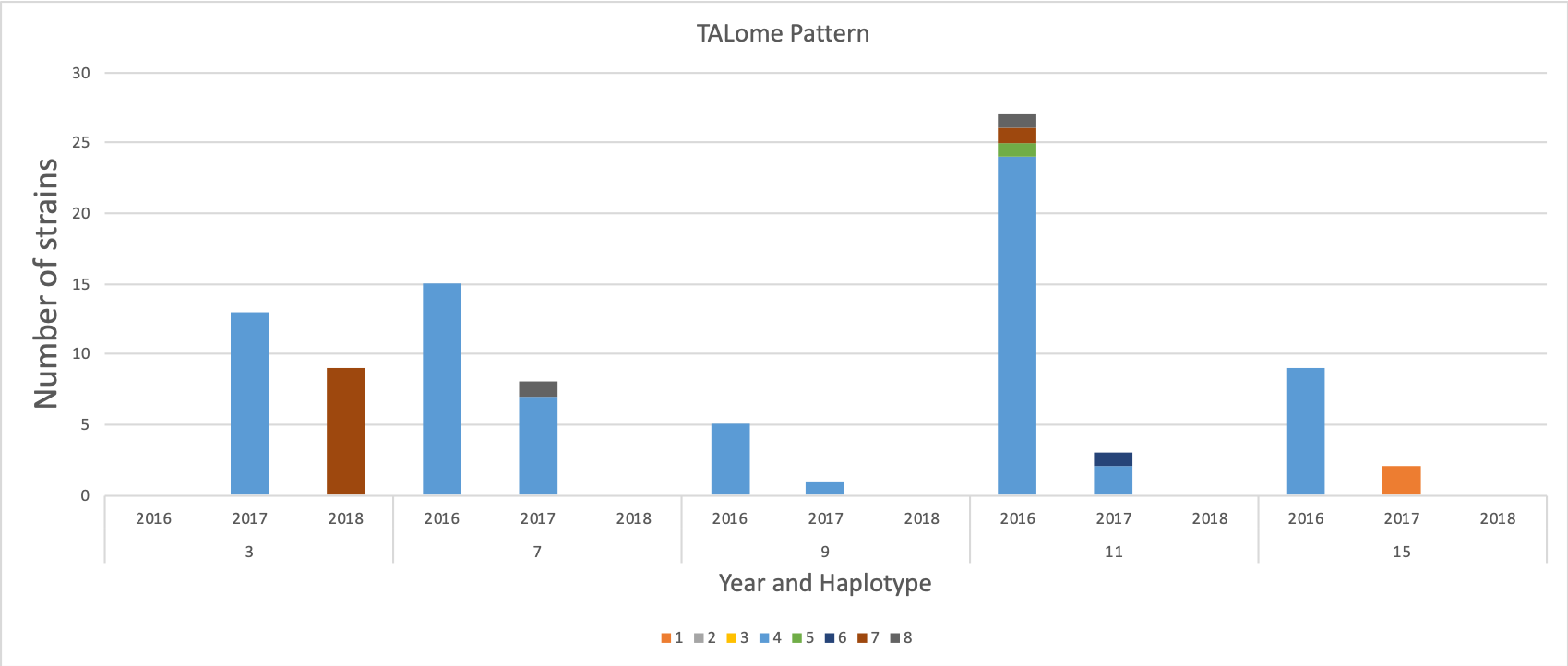

Supplement: Supplementary file 2 — Additional file 2: Table S2. MLVA scheme used and number of alleles at each locus [file 12284_2023_648_MOESM2_ESM.png]
