# Supplementary material for: Genetic Structure and TALome Analysis Highlight a High Level of Diversity in Burkinabe Xanthomonas Oryzae pv. oryzae Populations
Source: Rice (N Y). 2023 Jul 31;16:33. doi: 10.1186/s12284-023-00648-x (PMC10390441; doi:10.1186/s12284-023-00648-x)
Supplement: Supplementary file 3 — Additional file 3: Table S3. PCR condition and dilution used for genotyping [file 12284_2023_648_MOESM3_ESM.docx]

| Strain | Haplotype | TALome pattern | Field | Locality | Area | Year of isolation | Race | Field Group |
| --- | --- | --- | --- | --- | --- | --- | --- | --- |
| B2E10 | 3 | 4 | 2 | Bagré | Centre-Est | 2017 |  | 1 |
| B2E15 | 15 | 1 | 2 | Bagré | Centre-Est | 2017 | A3 | 1 |
| B2E16 | 15 | 1 | 2 | Bagré | Centre-Est | 2017 | A3 | 1 |
| B2E19 | 7 | 8 | 2 | Bagré | Centre-Est | 2017 |  | 1 |
| B2E2 | 3 | 4 | 2 | Bagré | Centre-Est | 2017 |  | 1 |
| B2E20 | 3 | 4 | 2 | Bagré | Centre-Est | 2017 |  | 1 |
| B2E22 | 7 | 4 | 2 | Bagré | Centre-Est | 2017 |  | 1 |
| B2E23 | 6 | 4 | 2 | Bagré | Centre-Est | 2017 |  | 1 |
| B2E24 | 3 | 4 | 2 | Bagré | Centre-Est | 2017 |  | 1 |
| B2E28 | 7 | 4 | 2 | Bagré | Centre-Est | 2017 |  | 1 |
| B2E35 | 7 | 4 | 2 | Bagré | Centre-Est | 2017 |  | 1 |
| B2E6 | 3 | 4 | 2 | Bagré | Centre-Est | 2017 |  | 1 |
| B2E9 | 3 | 4 | 2 | Bagré | Centre-Est | 2017 |  | 1 |
| B3E1 | 7 | 4 | 3 | Bagré | Centre-Est | 2017 |  | 1 |
| B3E15 | 3 | 4 | 3 | Bagré | Centre-Est | 2017 |  | 1 |
| B3E17 | 4 | 4 | 3 | Bagré | Centre-Est | 2017 |  | 1 |
| B3E18 | 3 | 4 | 3 | Bagré | Centre-Est | 2017 |  | 1 |
| B3E2 | 3 | 4 | 3 | Bagré | Centre-Est | 2017 |  | 1 |
| B3E24 | 7 | 4 | 3 | Bagré | Centre-Est | 2017 |  | 1 |
| B3E3 | 3 | 4 | 3 | Bagré | Centre-Est | 2017 | A3 | 1 |
| B3E4 | 3 | 4 | 3 | Bagré | Centre-Est | 2017 |  | 1 |
| B3E6 | 3 | 4 | 3 | Bagré | Centre-Est | 2017 |  | 1 |
| B4E10 | 17 | 4 | 4 | Bagré | Centre-Est | 2017 |  | 1 |
| B4E12 | 17 | 4 | 4 | Bagré | Centre-Est | 2017 |  | 1 |
| B4E13 | 17 | 4 | 4 | Bagré | Centre-Est | 2017 |  | 1 |
| B4E20 | 17 | 4 | 4 | Bagré | Centre-Est | 2017 |  | 1 |
| B4E23 | 3 | 4 | 4 | Bagré | Centre-Est | 2017 |  | 1 |
| B4E25 | 17 | 4 | 4 | Bagré | Centre-Est | 2017 |  | 1 |
| B4E28 | 7 | 4 | 4 | Bagré | Centre-Est | 2017 |  | 1 |
| B4E6 | 7 | 4 | 4 | Bagré | Centre-Est | 2017 |  | 1 |
| B4E7 | 17 | 4 | 4 | Bagré | Centre-Est | 2017 |  | 1 |
| B4E8 | 17 | 4 | 4 | Bagré | Centre-Est | 2017 |  | 1 |
| B6E2 | 11 | 6 | 4 | Bagré | Centre-Est | 2017 | A3 | 1 |
| B6E3 | 12 | 4 | 6 | Bagré | Centre-Est | 2017 |  | 2 |
| B6E4 | 11 | 4 | 6 | Bagré | Centre-Est | 2017 |  | 2 |
| B6E5 | 9 | 4 | 6 | Bagré | Centre-Est | 2017 |  | 2 |
| B6E7 | 11 | 4 | 6 | Bagré | Centre-Est | 2017 |  | 2 |
| BAI111 | 15 | 3 |  | Bagré | Centre-Est | 2012 | A3 |  |
| BAI145 | 21 | 2 | 5 | Di | Boucle du Mouhoun | 2016 | A3 | 5 |
| BAI146 | 21 | 2 | 5 | Di | Boucle du Mouhoun | 2016 | A3 | 5 |
| BAI147 | 21 | 2 | 5 | Di | Boucle du Mouhoun | 2016 |  | 5 |
| BAI148 | 21 | 2 | 5 | Di | Boucle du Mouhoun | 2016 |  | 5 |
| BAI149 | 21 | 2 | 5 | Di | Boucle du Mouhoun | 2016 |  | 5 |
| BAI150 | 21 | 2 | 5 | Di | Boucle du Mouhoun | 2016 |  | 5 |
| BAI152 | 21 | 2 | 5 | Di | Boucle du Mouhoun | 2016 |  | 5 |
| BAI153 | 21 | 2 | 5 | Di | Boucle du Mouhoun | 2016 |  | 5 |
| BAI154 | 21 | 2 | 5 | Di | Boucle du Mouhoun | 2016 |  | 5 |
| BAI155 | 21 | 2 | 5 | Di | Boucle du Mouhoun | 2016 |  | 5 |
| BAI156 | 21 | 2 | 5 | Di | Boucle du Mouhoun | 2016 |  | 5 |
| BAI157 | 21 | 2 | 5 | Di | Boucle du Mouhoun | 2016 |  | 5 |
| BAI158 | 21 | 2 | 5 | Di | Boucle du Mouhoun | 2016 |  | 5 |
| BAI159 | 21 | 2 | 5 | Di | Boucle du Mouhoun | 2016 |  | 5 |
| BAI160 | 21 | 2 | 5 | Di | Boucle du Mouhoun | 2016 |  | 5 |
| BAI161 | 21 | 2 | 5 | Di | Boucle du Mouhoun | 2016 |  | 5 |
| BAI163 | 21 | 2 | 5 | Di | Boucle du Mouhoun | 2016 |  | 5 |
| BAI164 | 21 | 2 | 5 | Di | Boucle du Mouhoun | 2016 |  | 5 |
| BAI165 | 21 | 2 | 5 | Di | Boucle du Mouhoun | 2016 |  | 5 |
| BAI166 | 20 | 2 | 10 | Di | Boucle du Mouhoun | 2016 | A3 | 6 |
| BAI167 | 20 | 2 | 10 | Di | Boucle du Mouhoun | 2016 | A3 | 6 |
| BAI168 | 20 | 2 | 10 | Di | Boucle du Mouhoun | 2016 | A3 | 6 |
| BAI169 | 20 | 2 | 10 | Di | Boucle du Mouhoun | 2016 | A3 | 6 |
| BAI170 | 20 | 2 | 10 | Di | Boucle du Mouhoun | 2016 | A3 | 6 |
| BAI171 | 20 | 2 | 10 | Di | Boucle du Mouhoun | 2016 |  | 6 |
| BAI172 | 20 | 2 | 10 | Di | Boucle du Mouhoun | 2016 |  | 6 |
| BAI173 | 20 | 2 | 10 | Di | Boucle du Mouhoun | 2016 |  | 6 |
| BAI174 | 20 | 2 | 10 | Di | Boucle du Mouhoun | 2016 |  | 6 |
| BAI175 | 20 | 2 | 10 | Di | Boucle du Mouhoun | 2016 |  | 6 |
| BAI176 | 20 | 2 | 10 | Di | Boucle du Mouhoun | 2016 |  | 6 |
| BAI177 | 20 | 2 | 10 | Di | Boucle du Mouhoun | 2016 |  | 6 |
| BAI178 | 20 | 2 | 10 | Di | Boucle du Mouhoun | 2016 |  | 6 |
| BAI180 | 20 | 2 | 10 | Di | Boucle du Mouhoun | 2016 |  | 6 |
| BAI181 | 11 | 4 | 14 | Bagré | Centre-Est | 2016 | A3 | 3 |
| BAI182 | 11 | 4 | 14 | Bagré | Centre-Est | 2016 | A3 | 3 |
| BAI183 | 11 | 4 | 14 | Bagré | Centre-Est | 2016 | A3 | 3 |
| BAI184 | 11 | 4 | 14 | Bagré | Centre-Est | 2016 |  | 3 |
| BAI185 | 11 | 4 | 14 | Bagré | Centre-Est | 2016 |  | 3 |
| BAI186 | 11 | 4 | 14 | Bagré | Centre-Est | 2016 |  | 3 |
| BAI187 | 15 | 4 | 14 | Bagré | Centre-Est | 2016 |  | 3 |
| BAI188 | 9 | 4 | 14 | Bagré | Centre-Est | 2016 |  | 3 |
| BAI189 | 11 | 7 | 14 | Bagré | Centre-Est | 2016 | A3 | 3 |
| BAI190 | 9 | 4 | 14 | Bagré | Centre-Est | 2016 |  | 3 |
| BAI191 | 9 | 4 | 14 | Bagré | Centre-Est | 2016 |  | 3 |
| BAI192 | 11 | 8 | 16 | Bagré | Centre-Est | 2016 | A3 | 4 |
| BAI193 | 7 | 4 | 16 | Bagré | Centre-Est | 2016 |  | 4 |
| BAI194 | 10 | 4 | 16 | Bagré | Centre-Est | 2016 |  | 4 |
| BAI195 | 11 | 4 | 16 | Bagré | Centre-Est | 2016 |  | 4 |
| BAI196 | 11 | 4 | 16 | Bagré | Centre-Est | 2016 | A3 | 4 |
| BAI197 | 11 | 4 | 16 | Bagré | Centre-Est | 2016 | A3 | 4 |
| BAI198 | 7 | 4 | 16 | Bagré | Centre-Est | 2016 |  | 4 |
| BAI199 | 8 | 4 | 16 | Bagré | Centre-Est | 2016 |  | 4 |
| BAI2 | 1 | 1 |  | Bagré | Centre-Est | 2003 | A1 |  |
| BAI200 | 11 | 4 | 16 | Bagré | Centre-Est | 2016 |  | 4 |
| BAI201 | 11 | 4 | 16 | Bagré | Centre-Est | 2016 |  | 4 |
| BAI202 | 9 | 4 | 17 | Bagré | Centre-Est | 2016 | A3 | 4 |
| BAI203 | 11 | 4 | 17 | Bagré | Centre-Est | 2016 |  | 4 |
| BAI204 | 11 | 4 | 17 | Bagré | Centre-Est | 2016 | A3 | 4 |
| BAI205 | 11 | 4 | 17 | Bagré | Centre-Est | 2016 |  | 4 |
| BAI206 | 9 | 4 | 17 | Bagré | Centre-Est | 2016 |  | 4 |
| BAI207 | 8 | 4 | 17 | Bagré | Centre-Est | 2016 |  | 4 |
| BAI208 | 15 | 4 | 17 | Bagré | Centre-Est | 2016 |  | 4 |
| BAI209 | 11 | 4 | 17 | Bagré | Centre-Est | 2016 |  | 4 |
| BAI210 | 11 | 4 | 17 | Bagré | Centre-Est | 2016 |  | 4 |
| BAI211 | 7 | 4 | 17 | Bagré | Centre-Est | 2016 |  | 4 |
| BAI212 | 5 | 4 | 17 | Bagré | Centre-Est | 2016 |  | 4 |
| BAI213 | 8 | 4 | 17 | Bagré | Centre-Est | 2016 |  | 4 |
| BAI215 | 11 | 4 | 17 | Bagré | Centre-Est | 2016 |  | 4 |
| BAI216 | 7 | 4 | 17 | Bagré | Centre-Est | 2016 | A3 | 4 |
| BAI217 | 2 | 4 | 13 | Bagré | Centre-Est | 2016 |  | 3 |
| BAI219 | 19 | 4 | 13 | Bagré | Centre-Est | 2016 |  | 3 |
| BAI220 | 11 | 4 | 13 | Bagré | Centre-Est | 2016 | A3 | 3 |
| BAI221 | 2 | 4 | 13 | Bagré | Centre-Est | 2016 | A3 | 3 |
| BAI222 | 11 | 4 | 13 | Bagré | Centre-Est | 2016 | A3 | 3 |
| BAI223 | 15 | 4 | 13 | Bagré | Centre-Est | 2016 |  | 3 |
| BAI224 | 19 | 4 | 13 | Bagré | Centre-Est | 2016 |  | 3 |
| BAI225 | 15 | 4 | 13 | Bagré | Centre-Est | 2016 |  | 3 |
| BAI226 | 15 | 4 | 13 | Bagré | Centre-Est | 2016 |  | 3 |
| BAI227 | 11 | 4 | 13 | Bagré | Centre-Est | 2016 |  |  |
| BAI228 | 11 | 4 | 13 | Bagré | Centre-Est | 2016 |  | 3 |
| BAI229 | 2 | 4 | 13 | Bagré | Centre-Est | 2016 |  | 3 |
| BAI230 | 19 | 4 | 13 | Bagré | Centre-Est | 2016 |  | 3 |
| BAI231 | 11 | 4 | 13 | Bagré | Centre-Est | 2016 |  | 3 |
| BAI232 | 19 | 4 | 13 | Bagré | Centre-Est | 2016 |  | 3 |
| BAI233 | 15 | 4 | 13 | Bagré | Centre-Est | 2016 |  | 3 |
| BAI234 | 15 | 4 | 13 | Bagré | Centre-Est | 2016 |  | 3 |
| BAI235 | 7 | 4 | 4 | Bagré | Centre-Est | 2016 | A3 | 1 |
| BAI236 | 7 | 4 | 4 | Bagré | Centre-Est | 2016 | A3 | 1 |
| BAI237 | 7 | 4 | 4 | Bagré | Centre-Est | 2016 | A3 | 1 |
| BAI238 | 7 | 4 | 4 | Bagré | Centre-Est | 2016 |  | 1 |
| BAI239 | 7 | 4 | 4 | Bagré | Centre-Est | 2016 |  | 1 |
| BAI24 | 1 | 1 |  | Bagré | Centre-Est | 2009 |  |  |
| BAI240 | 7 | 4 | 4 | Bagré | Centre-Est | 2016 |  | 1 |
| BAI241 | 7 | 4 | 4 | Bagré | Centre-Est | 2016 |  | 1 |
| BAI242 | 7 | 4 | 4 | Bagré | Centre-Est | 2016 |  | 1 |
| BAI243 | 13 | 4 | 4 | Bagré | Centre-Est | 2016 |  | 1 |
| BAI244 | 13 | 4 | 4 | Bagré | Centre-Est | 2016 |  | 1 |
| BAI245 | 7 | 4 | 4 | Bagré | Centre-Est | 2016 |  | 1 |
| BAI246 | 7 | 4 | 4 | Bagré | Centre-Est | 2016 |  | 1 |
| BAI247 | 11 | 4 | 4 | Bagré | Centre-Est | 2016 |  | 1 |
| BAI248 | 7 | 4 | 4 | Bagré | Centre-Est | 2016 |  | 1 |
| BAI250 | 11 | 5 | 15 | Bagré | Centre-Est | 2016 | A3 | 3 |
| BAI251 | 10 | 4 | 15 | Bagré | Centre-Est | 2016 |  | 3 |
| BAI252 | 8 | 4 | 15 | Bagré | Centre-Est | 2016 |  | 3 |
| BAI253 | 15 | 4 | 15 | Bagré | Centre-Est | 2016 |  | 3 |
| BAI254 | 11 | 4 | 15 | Bagré | Centre-Est | 2016 |  | 3 |
| BAI255 | 16 | 4 | 15 | Bagré | Centre-Est | 2016 | A3 | 3 |
| BAI257 | 18 | 4 | 15 | Bagré | Centre-Est | 2016 | A3 | 3 |
| BAI258 | 8 | 4 | 15 | Bagré | Centre-Est | 2016 |  | 3 |
| BAI259 | 10 | 4 | 15 | Bagré | Centre-Est | 2016 |  | 3 |
| BAI26 | 1 | 1 |  | Bagré | Centre-Est | 2009 |  |  |
| BAI261 | 15 | 4 | 15 | Bagré | Centre-Est | 2016 |  | 3 |
| BAI28 | 1 | 1 |  | Bagré | Centre-Est | 2009 | A10 |  |
| BAI29 | 1 | 1 |  | Bagré | Centre-Est | 2009 |  |  |
| BAI3 | 1 | 1 |  | Bagré | Centre-Est | 2003 | A10 |  |
| BAI30 | 1 | 1 |  | Bagré | Centre-Est | 2009 |  |  |
| BAI31 | 1 | 1 |  | Bagré | Centre-Est | 2009 |  |  |
| BAI33 | 1 | 1 |  | Bagré | Centre-Est | 2009 | A10 |  |
| BAI45 | 1 | 1 |  | Niassan | Boucle du Mouhoun | 2011 |  |  |
| BAI50 | 1 | 1 |  | Niassan | Boucle du Mouhoun | 2011 | A10 |  |
| BAI53 | 1 | 1 |  | Niassan | Boucle du Mouhoun | 2011 |  |  |
| BAI54 | 1 | 1 |  | Niassan | Boucle du Mouhoun | 2011 |  |  |
| BAI55 | 1 | 1 |  | Niassan | Boucle du Mouhoun | 2011 | A10 |  |
| BAI56 | 1 | 1 |  | Niassan | Boucle du Mouhoun | 2011 |  |  |
| BAI57 | 1 | 1 |  | Niassan | Boucle du Mouhoun | 2011 |  |  |
| BAI58 | 1 | 1 |  | Niassan | Boucle du Mouhoun | 2011 |  |  |
| BAI59 | 1 | 1 |  | Niassan | Boucle du Mouhoun | 2011 |  |  |
| BV1-9 | 3 | 7 | 2 | Bagré | Centre-Est | 2018 |  | 1 |
| BV2-1 | 3 | 7 | 2 | Bagré | Centre-Est | 2018 |  | 1 |
| BV2-3 | 3 | 7 | 2 | Bagré | Centre-Est | 2018 |  | 1 |
| BV2-8 | 3 | 7 | 2 | Bagré | Centre-Est | 2018 |  | 1 |
| BV3-9 | 3 | 7 | 2 | Bagré | Centre-Est | 2018 |  | 1 |
| BV5-2 | 3 | 7 | 2 | Bagré | Centre-Est | 2018 |  | 1 |
| BV5-6 | 3 | 7 | 2 | Bagré | Centre-Est | 2018 |  | 1 |
| BV5-7 | 3 | 7 | 2 | Bagré | Centre-Est | 2018 |  | 1 |
| BV5-8 | 3 | 7 | 2 | Bagré | Centre-Est | 2018 |  | 1 |
| M3-4 | 14 | 2 | 2 | Mogtédo | Centre-Est | 2018 | A3 | 1 |
| V10-1 | 22 | 2 | 1 | Di | Boucle du Mouhoun | 2018 |  | 7 |
| V10-4 | 22 | 2 | 1 | Di | Boucle du Mouhoun | 2018 |  | 7 |
