# Supplementary material for: Genetic Structure and TALome Analysis Highlight a High Level of Diversity in Burkinabe Xanthomonas Oryzae pv. oryzae Populations
Source: Rice (N Y). 2023 Jul 31;16:33. doi: 10.1186/s12284-023-00648-x (PMC10390441; doi:10.1186/s12284-023-00648-x)
Supplement: Supplementary file 4 — Additional file: Fig. S1 Rarefaction curve of genotypes obtained with the MLVA-14 scheme on the 177 Xoo strains from Burkina Faso. The four monomorphic loci were removed of this analysis [file 12284_2023_648_MOESM4_ESM.docx]

|  |  | Motif length (bp) | Na^1^ |
| --- | --- | --- | --- |
|  |  |  |  |
| Mix 1 | G06 | 6 | 3 |
|  | G07 | 7 | 7 |
|  | G58 | 4&7 | - |
|  | G81 | 6 | 4 |
| Mix 2 | G2553 | 8 | 7 |
|  | G88 |  | - |
|  | G44 | 7 | 2 |
|  | G62 | 5 | 2 |
| Mix 3 | G55 | 8 | 8 |
|  | G59 | 6 | 4 |
|  | G60 | 6 | 4 |
|  | G67 | 7 | 3 |
| Mix 4 | G09 | 7 | 1 |
|  | G15 | 7 | 1 |
|  | G80 | 7 | 1 |
|  | G83 | 5 | 1 |

^1^ Na number of alleles estimated from the whole African *Xoo* collection (n = 177).
