# Supplementary material for: Genetic Structure and TALome Analysis Highlight a High Level of Diversity in Burkinabe Xanthomonas Oryzae pv. oryzae Populations
Source: Rice (N Y). 2023 Jul 31;16:33. doi: 10.1186/s12284-023-00648-x (PMC10390441; doi:10.1186/s12284-023-00648-x)
Supplement: Supplementary file 5 — Additional file: Fig. S2 Distribution of TALome profiles in haplotypes containing Xoo strains collected in Bagr? between 2016 and 2018 [file 12284_2023_648_MOESM5_ESM.docx]

| Mix 1 & 2 | 95°C | 15min |  |
| --- | --- | --- | --- |
|  | 94°C | 30s | x 35 |
|  | 60°C | 1min30 |  |
|  | 72°C | 1min |  |
|  | 72°C | 20min |  |
|  | 14°C | ∞ |  |
|  | dilution 1/150 | | |
| Mix 3 & 4 | 95°C | 15min |  |
|  | 94°C | 30s | x 25 |
|  | 64°C | 1min30 |  |
|  | 72°C | 1min |  |
|  | 60°C | 20min |  |
|  | 14°C | ∞ |  |
|  | dilution 1/75 | | |
